# Virus Inactivation in Water Using Laser-Induced Graphene Filters

**DOI:** 10.3390/ma14123179

**Published:** 2021-06-09

**Authors:** Najmul Haque Barbhuiya, Swatantra P. Singh, Arik Makovitzki, Pradnya Narkhede, Ziv Oren, Yaakov Adar, Edith Lupu, Lilach Cherry, Arik Monash, Christopher J. Arnusch

**Affiliations:** 1 Environmental Science and Engineering Department (ESED), Indian Institute of Technology Bombay, Mumbai 400076, India; najmul@iitb.ac.in; 2 Centre for Research in Nanotechnology & Science (CRNTS), Indian Institute of Technology Bombay, Mumbai 400076, India; 3 Department of Biotechnology, Israel Institute for Biological Research, Ness Tiona 7410001, Israel; arikm@iibr.gov.il (A.M.); zivo@iibr.gov.il (Z.O.); yaakova@iibr.gov.il (Y.A.); edithl@iibr.gov.il (E.L.); lilachc@iibr.gov.il (L.C.); arikmo@iibr.gov.il (A.M.); 4 Albert Katz International School for Desert Studies, The Jacob Blaustein Institutes for Desert Research, Ben-Gurion University of the Negev, Sede-Boqer Campus 8499000, Israel; pradnya.n.n@gmail.com; 5 Department of Desalination and Water Treatment, The Zuckerberg Institute for Water Research, The Jacob Blaustein Institutes for Desert Research, Ben-Gurion University of the Negev, Sede Boqer Campus 8499000, Israel

**Keywords:** laser-induced graphene, antiviral, antibacterial, disinfection, conductive filters

## Abstract

Interest in the pathogenesis, detection, and prevention of viral infections has increased broadly in many fields of research over the past year. The development of water treatment technology to combat viral infection by inactivation or disinfection might play a key role in infection prevention in places where drinking water sources are biologically contaminated. Laser-induced graphene (LIG) has antimicrobial and antifouling surface effects mainly because of its electrochemical properties and texture, and LIG-based water filters have been used for the inactivation of bacteria. However, the antiviral activity of LIG-based filters has not yet been explored. Here we show that LIG filters also have antiviral effects by applying electrical potential during filtration of the model prototypic poxvirus *Vaccinia lister*. This antiviral activity of the LIG filters was compared with its antibacterial activity, which showed that higher voltages were required for the inactivation of viruses compared to that of bacteria. The generation of reactive oxygen species, along with surface electrical effects, played a role in the mechanism of virus inactivation. This new property of LIG highlights its potential for use in water and wastewater treatment for the electrochemical disinfection of various pathogenic microorganisms, including bacteria and viruses.

## 1. Introduction

Worldwide, viral infections alone claim nearly 15 million human lives annually [1]. Vaccination is considered to be an effective preventive measure against viral infection. However, the lack of effective vaccines against different viruses and their variants, combined with the lag time for their development and testing, requires other protection measures for the prevention of viral transmission [2]. Thus, the development of technology that inactivates viruses and bacteria in water and air will prevent viable viruses from entering the host and can help in combating viral infections. Viruses are generally smaller than bacteria, and they consist of viral nucleic acid protected by a proteinaceous layer called the capsid. In some viruses, the capsid can be enclosed by a protective envelope. According to the World Health Organization, many water-transmitted viruses, including adenoviruses, enteroviruses, rotavirus, astrovirus, and hepatitis A and E viruses, pose a moderate to high health risk [3]. Some viruses are also excreted through excreta and have the potential to spread via water [3,4]. Water can also transmit viruses, for example, via skin and eye contact while swimming or via inhalation while showering, causing ocular and respiratory infections [5]. Conventional wastewater treatment plants can partially remove viruses, but the safe disposal or reuse of effluent highly depends on the final disinfection efficacy [6,7,8,9]. Thus, virus disinfection in wastewater might inhibit the indirect infection pathways [6] and might increase the safety of wastewater reuse. Membrane filtration has the potential to provide a barrier to virus passage; however, electrically conductive surfaces on water filters including graphene can lead to electrochemical surface effects that have been shown to inactivate bacteria. These effects might also cause virus inactivation or removal with no toxic byproduct formation [10].

Various types of carbon-based materials, including carbon nanotubes, carbon nanospheres, graphene, and their derivatives, have been widely used for water and wastewater treatment [11,12,13]. Emerging materials like metal–organic frameworks (MOFs) and LIG can be used to form thin films and have shown promising results for their application in various environmental applications [14,15,16]. Among the various materials, graphene is considered to have unique and superior properties. Graphene consists of a single layer of sp^2^-hybridized carbon atoms arranged in a hexagonal lattice [17,18]. Due to its extraordinary physicochemical properties, graphene and related derivatives have been used in various applications, ranging from electronics to environmental remediation [19,20,21]. Recently, researchers demonstrated a novel, cost-effective, and scalable technique for fabricating graphene coatings on various polymeric materials via laser scribing, called laser-induced graphene (LIG) [22,23]. Certain materials suitable for LIG formation are commonly used in membrane filtration [22,23,24], and the antibacterial and antibiofilm properties of LIG have been explored for water and air filtration [16,24,25,26,27]. Thus, LIG might enhance water treatment technologies and water filtration, and in the present study, we investigate the ability of porous LIG membranes to inactivate viruses.

Many researchers have explored the various mechanisms by which graphene-based filters can inactivate or kill microorganisms. In the absence of electrical potential, graphene has been demonstrated to rupture cell membranes by inducing mechanical stress on direct contact [28,29,30,31,32,33,34], to sequester microbial cells [29,35,36,37,38,39], to cause instability in the cell system due to nanoscale dewetting [40,41,42,43,44], and to oxidize important cellular components [21,38,44,45,46,47,48,49,50,51], thereby exacerbating cellular death/inactivation. However, these mechanisms require considerable contact time for microbial inactivation and disinfection. On the other hand, in the presence of electrical potential, conductive filters can instantly remove, kill, or inactivate microorganisms by various mechanisms such as direct and indirect oxidation, Coulombic repulsion, bubble generation, Joule heating, and local pH change [52,53,54,55,56,57,58,59]. The electrical conductivity of the conductive filters might also contribute to lipid membrane disruption through the electroporation process [21,60,61,62]. Thus, the physicochemical and electrochemical manifold effects of graphene, including LIG filters, can potentially also be useful for the inactivation of viruses. Furthermore, the integration of renewable energy like solar energy with membrane processes can further make the use of LIG filters and membranes more sustainable [63].

In this study, we demonstrate the antiviral ability of LIG filters against the *Vaccinia lister* virus, a prototypic poxvirus, at different applied potentials. LIG filters were fabricated using polyethersulfone ultrafiltration membranes and used to test the inactivation of the virus by conducting plaque assays, and the destructive effects on the virion particles were observed via transmission electron microscopy (TEM) analysis. We discuss the possible mechanisms of LIG virion inactivation in comparison to LIG’s effects on bacteria. The results show that the LIG-based conductive filters are a promising candidate in providing high-efficiency viral/bacterial disinfection with low energy consumption, which can be explored in different water treatment and membrane filtration processes.

## 2. Materials and Methods

### 2.1. LIG Filter Fabrication

The LIG membrane filters were fabricated via a method similar to that described in our previous work [24]. Briefly, polyethersulfone (PES) (UP150, Microdyn-Nadir, Wiesbaden, Germany) membranes were lased with a focused laser spot using a VLS 3.50 (Universal Laser Systems, (Distributor: Caliber, Tel Aviv, Israel)) laser platform, which was equipped with a 10.6 μm CO_2_ pulse laser (50 W, 2.0 inch Lens Kit) (Universal Laser Systems, (Distributor: Caliber, Tel Aviv, Israel)). The LIG was obtained with a laser setting of 70 PPI image density, 25% scan rate, and 0.1% laser duty cycle, made in the presence of air under ambient conditions. The circular LIG filters were ~200 μm thick with a radius of 23 mm. The untreated membranes and the LIG filters were characterized by Raman spectroscopy, X-ray photoelectron spectroscopy (XPS), scanning electron microscopy (SEM), and contact angle measurements, both prior to and after laser modification, as previously described [24].

### 2.2. Antiviral and Antimicrobial Activity of LIG Filters

Carbon wires were attached to circular, LIG-coated PES filters using a carbon-based glue. Two LIG electrode filters were stacked one on top of the other and placed in a dead-end filtration unit (Figure S1). Each electrode was connected to a direct current (DC) voltage supply, and unless otherwise stated, the connections were made such that the anode was situated atop the cathode. A secondary connection was made to a multimeter such that the current through the electrodes could be simultaneously monitored. Bacterial testing was performed as previously described using a bacterial culture mix [24] For viral tests, *Vaccinia lister* in 30 mL phosphate buffer saline (PBS) was passed through the conductive LIG filters using a vacuum pump at a constant flow rate of ~1000 L m^−2^ h^−1^ in a dead-end filtration mode. Then, the filter was washed with an additional 30 mL PBS. The power was adjusted to 2.5, 5.0, 10.0, and 20.0 V for these filtration experiments. Control experiments in the absence of electrical potential were also performed. The feed and permeate were collected and serially diluted, and the microbes were enumerated using plaque assay for viruses and plate counting methods for bacteria [24].

#### 2.2.1. Plaque Assay of Vaccinia Virus-Infected Vero Cells

In a six-well plate (Costar Corning Incorporated, NY, USA), 7 × 10^5^ Vero cells (ATCC CCL-81) in suspension at a volume of 3 mL were plated for each well with DMEM medium supplemented with 10% heat-inactivated FCS (Biological Industries LTD, BET-Haemek, Israel). The cell culture was grown for one day at 37 °C, in 5% CO_2_, to a monolayer. The *Vaccinia* virus standard curve was diluted to an estimated concentration of 150–600 plaque-forming unit (PFU) mL^−1^. Then, 0.3 mL of the virus sample/standard was added to 0.2 mL of medium for each well. The plates were incubated with agitation for 60 min at 37 °C, in 5% CO_2_, with a relative humidity of > 85%. The medium was removed, and 1 mL of 0.1% crystal-violet solution was added and incubated at room temperature for 5 min. The solution was removed, and the cells were washed with water. The plaques were counted manually.

#### 2.2.2. Negative Staining Transmission Electron Microscopy

The drop-on-the-grid method (DEG) was used as follows. A drop (10 µL) of sample suspension containing 10^5^ Vaccinia viruses was fixated by incubation with 2.5% paraformaldehyde (Electron Microscopy Sciences, PA, USA) for 30 min at rt followed by another 30 min of incubation at 37 °C. Then, the sample was placed directly onto a glow-discharged EM sample support, 300 MESH copper grid, covered with carbon film (Electron Microscopy Sciences, PA, USA). After adsorption for 10 min at room temperature, the grid was washed three times in double-distilled water and negatively stained with 1% phosphotungstic acid, pH 4.5. The grids were examined using a TECNAI T12 FEI (ThermoFisher, OR, USA) transmission electron microscope operated at 200 kV. Micrographs were recorded using an Erlangsheng 782 ES 500W camera (Gatan, CA, USA) at a resolution of 2048 × 2048 pixels.

#### 2.2.3. H_2_O_2_ and Free Chlorine Generation Test

The electrogeneration of H_2_O_2_ was tested with aqueous solutions of NaCl (0.9% *w*/*w*) and Na_2_SO_4_ (0.9% *w*/*w*). Each solution was passed through the stacked LIG filters under the application of various voltages (2.5 V, 5 V, 10 V, and 20 V) with ~1000 L m^−2^ h^−1^ flow rate. The concentrations of H_2_O_2_ and free chlorine in the permeate were quantified using the DMP and DPD methods, respectively [64].

## 3. Results and Discussion

**Figure 1:**
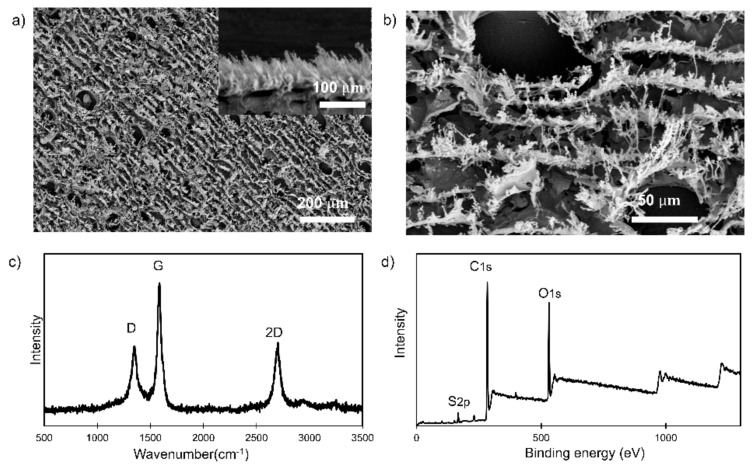
(**a**) Scanning electron microscopy (SEM) image of an laser-induced graphene (LIG) filter at low resolution (Inset: Cross-sectional SEM image of an LIG filter); (**b**) SEM image of an LIG filter at high resolution (Scale: 50 µm); (**c**) Raman spectrum for an LIG filter; (**d**) X-ray photoelectron spectroscopy (XPS) survey for an LIG filter.

The LIG filters using PES membranes as substrates were fabricated according to the method used in our previous study [24]. The filters were characterized using SEM, XPS, and Raman spectroscopy. The SEM images showed a highly porous structure for LIG filters (Figure 1a,b) and a characteristic porous foamlike structure in the cross section (Figure 1a, inset). Contact angle measurements were made for unused (39.7 ± 1.1°) and used LIG filters (30.5 ± 2.4° for the anodic and 28.4 ± 0.9° for the cathodic filter). The used cathodic and anodic LIG filters were slightly more hydrophilic than the unused filters and suggested that oxidation of the LIG might occur during filtration (Figure S2). XPS revealed the chemical composition information of the material surface (Figure 1c). It showed C 1s, O 1s, and S 2p peaks at ~285.5, ~534, and ~167 eV, respectively, which was also similar to the results of previous studies [24,65,66]. This confirmed the presence of C–S bonding in the LIG skeleton, which was absent in the PES membrane substrate. The Raman spectra showed the characteristic D, G, and 2D peaks for graphene at ~1350 cm^−1^, 1580 cm^−1^, and 2700 cm^−1^, respectively (Figure 1d). A high degree of graphene formation and graphitization was supported by the D/G and 2D/G intensity ratios.

### 3.1. Antiviral Activity of the LIG Filters

**Figure 2:**
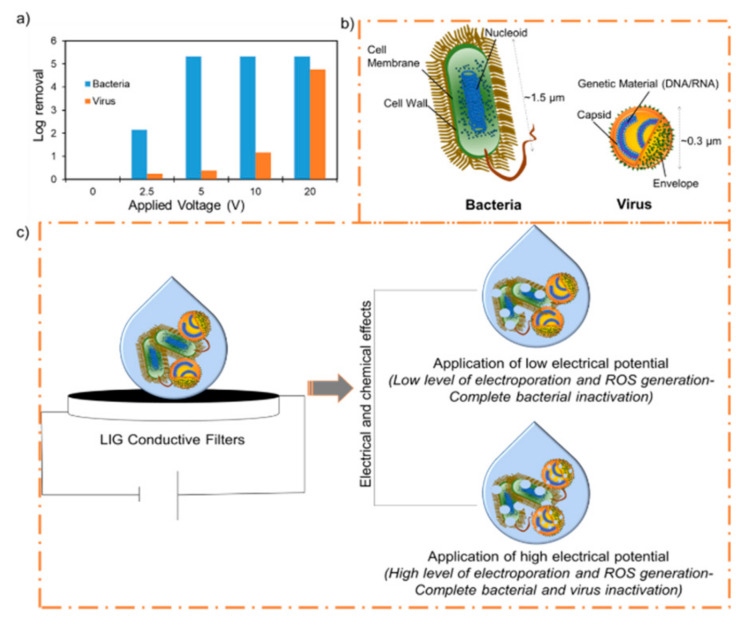
(**a**) Bacterial and virus inactivation at different voltages at a constant flow rate of ~1000 L m^−2^ h^−1^; (**b**) Comparison of the major structural components of the virion and bacteria that might be affected by the electrically active filters; (**c**) Proposed mechanism for microbial and viral inactivation.

The LIG filters in an anode-on-cathode stacked configuration were used with an externally applied DC voltage to test their antimicrobial or antiviral activity. The LIG filters were able to inactivate or kill bacteria and virions under the application of different applied voltages (Figure 2a).

**Figure 3:**
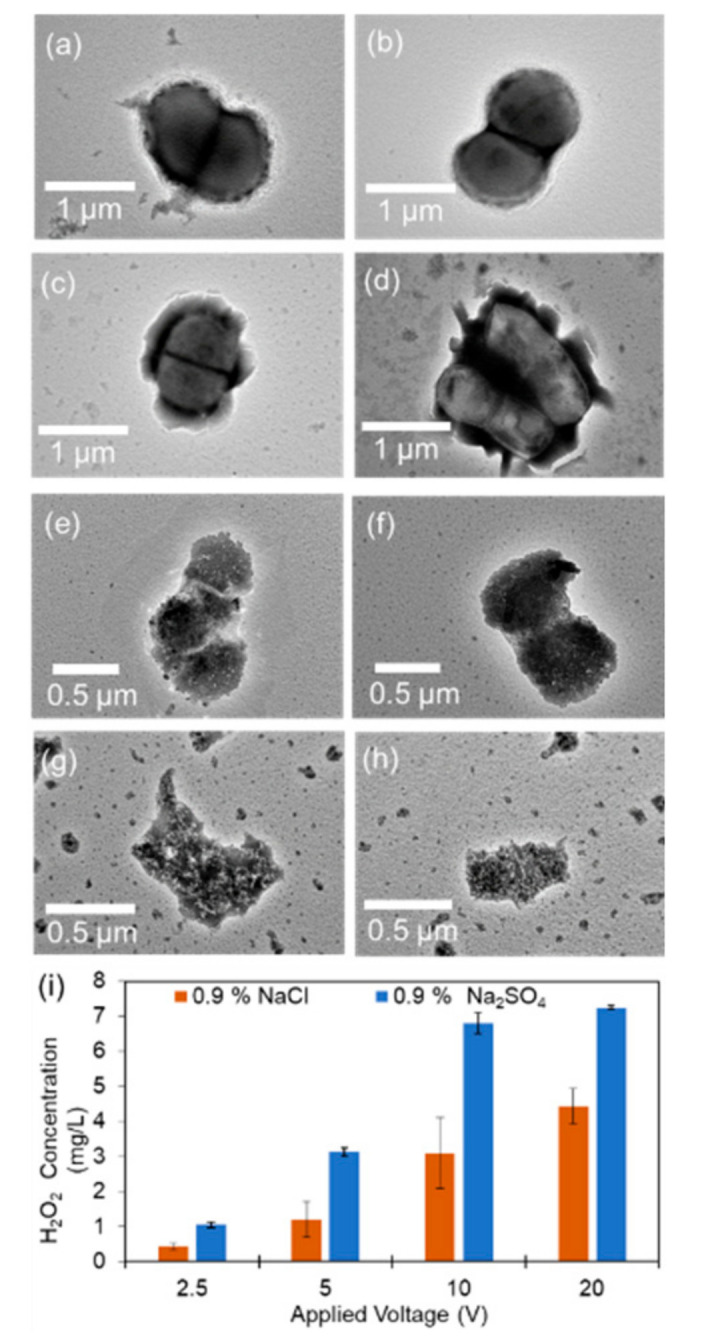
TEM images of the virions inactivated at different voltages: (**a**,**b**) 0 V; (**c**,**d**) 5 V; (**e**,**f**) 10 V; (**g**,**h**) 20 V; (**i**) H_2_O_2_ concentration in the permeate when 0.9% NaCl and 0.9% Na_2_SO_4_ were used as feed.

A virus-containing suspension was filtered through the stacked LIG filters at 2.5 V, 5.0 V, 10.0 V, and 20.0 V at ~1000 L m^−2^ h^−1^. Unfiltered suspensions maintained at room temperature during the course of each experiment were used as controls. The application of low electrical potentials was not effective in inactivating virions, as shown in Figure 2a and Figure 3. Low potentials of 2.5 V and 5 V did not cause much damage to the virions, and this was confirmed by the TEM images up to 5 V (Figure 3c,d).

At this voltage, 40–60% virus removal was seen, while the same conditions resulted in 100% bacterial removal (Figure S3). However, higher electrical potential (10 V) caused significant damage to viral particles (Figure 3e,f), and ~92% removal was observed. Further testing at 20 V resulted in complete inactivation (>4 log virus removal), and destruction of the virion was seen (Figure 3g,h). Virion damage might be because of similar mechanisms described for bacterial inactivation, including electrical and chemical effects [26]. However, virions are reported to be more resistant than bacteria towards chemical disinfectants such as chlorine and hydrogen peroxide [67,68,69], and this might explain the differences in the activity. H_2_O_2_ and chlorine generation were found to be voltage dependent [24,26,70], which would have increased with increasing voltage, resulting in more indirect oxidation of microbial cellular materials. Also, a higher electric field is required for the electroporation effect in smaller organisms [71], and this provides a plausible explanation for the relatively higher electrical potential required for viral destruction compared to bacterial destruction (Figure 2c). The transmembrane voltage induced in the cell membrane of the organism under an applied electric field is proportional to the size of the organism. Thus, a greater field is required for electroporation of smaller virus particles than for bacterial cells [62,71,72,73,74]. Moreover, since the number concentrations of bacteria and viruses, along with the flow rates, were approximately the same for both bacterial and virion inactivation, the lower inactivation of virions was also probably due to the smaller size of virions (Figure 2b). Here, less contact with the LIG would result in less direct electrical effects and lower charge transfer, and the indirect chemical effects might play a greater role in the mechanism, displayed by the increased voltage requirement for disinfection [71]. Additionally, an increase in electrical potential can increase the electrophoretic movement of the virions towards the anode, resulting in direct oxidation and increased virus removal; a similar effect was seen for bacterial cells. A detailed explanation of these mechanisms of microbial inactivation by LIG electrodes can be found elsewhere [26]. Briefly, the antimicrobial activity of the LIG filters increases when used as electrode pairs under electrical potentials. The microbial killing possibly involves physical destruction due to contact with the LIG, chemical oxidation, or the electrical effects of LIG electrodes. The movement of microbes towards LIG surfaces, combined with surface toxicity and the generation of localized active chemical species, is the most plausible antimicrobial action mechanism. Also, the concentration of active chemical species is higher near the electrodes than in the bulk solution. Thus, the electrostatic attraction of microbes towards the anode further exposes them to a higher concentration of active chemical species. The LIG electrodes may also cause physical damage to the microbe via irreversible electroporation or direct electron transfer [60,71,75,76]. Taken together, the various antimicrobial effects of conductive LIG filters are also useful for inactivating viruses, but at higher potentials.

The voltage-dependent microbicidal efficiency of LIG filters is consistent with the explanation that an increase in the applied potential over the electrodes leads to an associated increase in the electric field and current density. This causes microbial death via electroporation and mechanical destruction, and redox processes can occur that generate toxic chemical species, which can oxidize structural features of the microbes and cause oxidative-stress-mediated death [26]. Components in the lipid membrane and negatively charged lipids make bacteria anionic. Similarly, in virions, the outer capsid surfaces are generally also negatively charged at near-neutral pH values, and increasing the pH results in increased negative surface charge densities [77,78]. As a consequence, microbes are predicted to possess a Coulombic attraction to the positively charged anode while being repelled by the negatively charged cathode. This reasoning motivated the placement of the anode above the cathode in the constructed filtration system; the deformation and elongation of cells can occur as they are pulled against the irregular anode surface, thereby affecting the membrane rupture and death of the bacteria [26]. This electrostatically induced mechanical effect is expected to increase as the externally applied voltage is increased [26]. Similarly, the virion particles are destroyed at a higher voltage and can be seen in Figure 2e–h. To confirm that the anode-on-top configuration of LIG filters is most effective, the antimicrobial activity was evaluated as previously, except with the cathodic filter situated above the LIG anode. However, the effect was minor, as we observed that the cathode-on-top filter configuration was only slightly less effective at microbial killing (Table S1).

### 3.2. H_2_O_2_ and Free Chlorine Generation

The H_2_O_2_ and free chlorine concentrations were measured in the permeate of the stacked filters under 2.5 V, 5 V, 10 V, and 20 V. An increase in H_2_O_2_ concentration was observed with an increase in the applied voltage (Figure 3i). A maximum of ~5 mg L^−1^ of H_2_O_2_ was obtained at 20 V with 0.9% NaCl. However, ~7 mg L^−1^ of H_2_O_2_ was detected at 20 V with Na_2_SO_4_ (0.9%). This difference may be due to the generation of free chlorine at the anode with NaCl. The free chlorine can react with the H_2_O_2_, thus reducing its concentration [26,64]. However, it should be noted that no free chlorine was detected in the permeate for either solution. In other studies, solutions containing viruses and 5–7 mg L^−1^ H_2_O_2_ concentrations required hours for the observation of virus inactivation (< 0.1–1 log reduction of viruses) [79,80]. To achieve a 4 log or greater level of virus inactivation, higher doses of H_2_O_2_ or longer contact time was required [81,82,83,84]. One study reported the use of 7.5% H_2_O_2_ for 20 min for ~5 log level inactivation of vaccinia virus [85]. In our present study, we observed ~4 log removal with a lower concentration of H_2_O_2_ (~5 mg L^−1^) and with a shorter filtration time (~4 min). Thus, the electrochemical effect near the surface of the LIG filters might play an important role in the bacterial and viral inactivation.

### 3.3. Current–Voltage Relationships Present in LIG-PES Filters

The high surface area and intrinsic charge-carrier mobility of LIG filters allow high current densities from relatively low voltages, resulting in the rapid and energy-efficient killing of virions via electrical contact and the formation of redox-active chemical species such as H_2_O_2_ at the lower, cathodic filter and Cl_2_ at the upper, anodic filter, as seen in the following equations:

2 Cl^–^ (*aq*) → 2 Cl_2_ (*g*) + 2 *e*^–^ (1)

O_2_ (*g*) + 2 H^+^ (*aq*) + 2 *e*^–^ → H_2_O_2_ (*aq*) (2)

The current–voltage relationship for the LIG filters used during experimentation was elucidated using 0.9% NaCl solution (Table S2). As predicted, the extensive surface area of the LIG filters leads to the generation of large currents and can enhance electrochemical processes, even at low voltages [26].

## 4. Conclusions

Sulfur-doped LIG filters were prepared using PES membranes as substrates. The filters were characterized and were found to be highly porous and electrically conductive. The antiviral activity of the LIG filters was investigated under the application of electrical potential. A *Vaccinia* virus suspension was filtered through the stacked LIG filters under varied applied potential. The application of low potential was sufficient for 6 log removal of the bacteria; however, a higher potential application was required for 4 log removal of the virus. The virion damage may be due to electrochemical effects, including electroporation, reactive chlorine, and oxygen species generation. The higher electrical potential requirement is due to the smaller size of the virus and its greater resistance towards reactive chemical species like H_2_O_2_. LIG-integrated membranes and filters have the potential to build the next generation of cost-effective systems for simultaneous water filtration and disinfection of pathogenic microbes, including bacteria and viruses.

## Data Availability

Data is contained within the article or supplementary material or is available on request from the corresponding authors.

## Supplementary Materials

The following are available online at https://www.mdpi.com/article/10.3390/ma14123179/s1, Figure S1: Experimental setup for filtration experiments, Figure S2: Contact-angle of LIG-PES membrane surfaces, Figure S3: Comparative percentage removal of bacteria and viruses under the application of different electrical potential, Table S1: Bacterial inactivation at a fixed vacuum pressure and varying LIG electrode configurations, Table S2: Current-voltage relationship of LIG-PES electrodes.

## Supporting Information File

Experimental setup scheme, contact angle, bacterial/virus% removal, bacterial inactivation with different electrode configuration, IV relationship of LIG electrodes.
