# Supplementary material for: Virus Inactivation in Water Using Laser-Induced Graphene Filters
Source: Materials (Basel). 2021 Jun 9;14(12):3179. doi: 10.3390/ma14123179 (PMC8226673; doi:10.3390/ma14123179)
Supplement: Supplementary file 1 [file materials-14-03179-s001.zip › materials-1186332-supplementary.pdf]

materials

Supplementary Materials

Virus Inactivation in Water Using Laser-Induced Graphene Filters

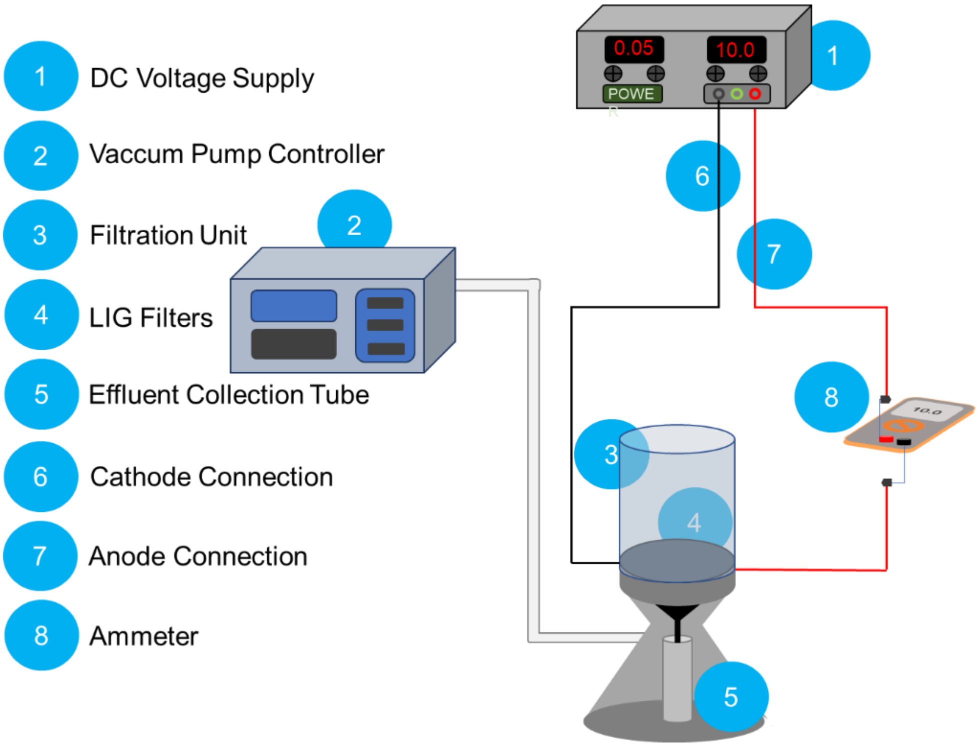

Figure S1. Experimental setup for filtration experiments.

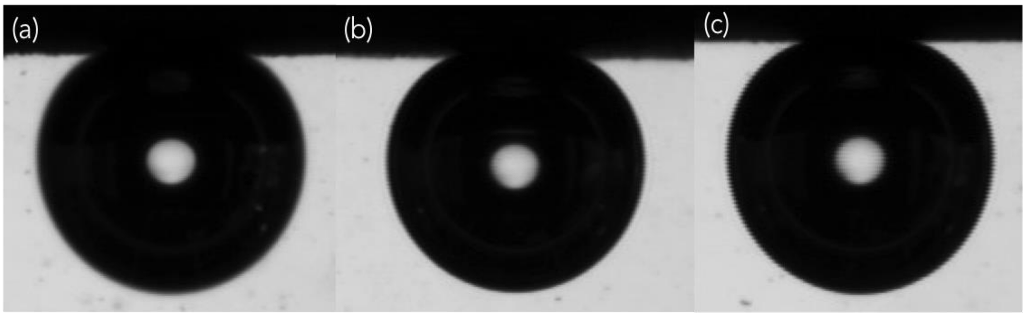

Figure S2. Contact-angle experiments using laser-induced graphene-poly(ethersulfone) (LIG-PES) membrane surfaces. (**a**) Unused LIG-PES electrode; the average contact angle was $\Theta = 39.7 \pm 1.1°$. (**b**) Used upper/anodic LIG-PES electrode; $\Theta = 30.5 \pm 2.4°$. (**c**) Used lower/cathodic LIG-PES electrode; $\Theta = 28.4 \pm 0.9°$.

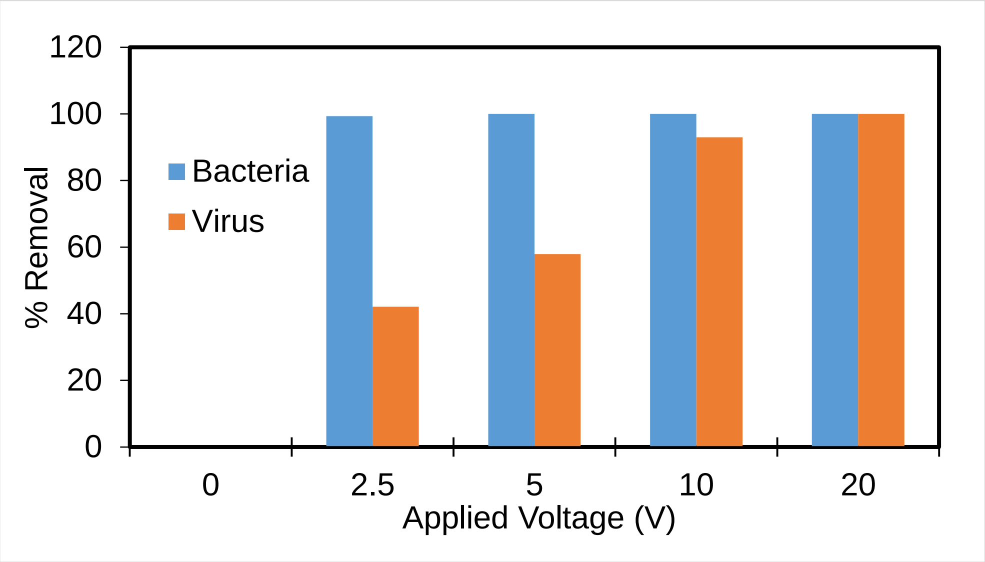

Figure S3. Comparative percentage removal of bacteria and viruses under the application of different electrical potential.

Table S1. Bacterial inactivation at a fixed vacuum pressure and varying laser-induced graphene (LIG) electrode configurations. Average flux with anode on top: 1810 L/m^2 h. Average flux with cathode on top: 1794 L/m^{2}h.

Electrode Configuration	Voltage (V)	Permeate Flux (L/m^2 h)	Percentage of Bacteria Killed	Log Removal
Anode-on-Top	2.5	1865	~98.7	1.9
	5.0	1722	> 99.99	> 4
	10.0	1865	> 99.99	> 4
	20.0	1790	> 99.99	> 4
Cathode-on-Top	2.5	1790	~95.4	1.3
	5.0	1658	> 99.99	> 4
	10.0	1865	> 99.99	> 4
	20.0	1865	> 99.99	> 4

Table S2. Current-voltage relationship of LIG-PES electrodes.

External Voltage (V)	Current (mA)
2.5	6.3
5.0	19.0
10.0	53.8
20.0	152.6
